# Genetic evidence for prevalence of alloparental care in a socially monogamous biparental cichlid fish, *Perissodus microlepis*, from Lake Tanganyika supports the “selfish shepherd effect” hypothesis

**DOI:** 10.1002/ece3.2089

**Published:** 2016-03-21

**Authors:** Hyuk Je Lee, Valentin Heim, Axel Meyer

**Affiliations:** ^1^ Chair in Zoology and Evolutionary BiologyDepartment of BiologyUniversity of Konstanz78457KonstanzGermany; ^2^ Department of Biological ScienceCollege of Science and EngineeringSangji UniversityWonju220‐702Korea

**Keywords:** Brood farming out, extra‐pair matings, maladaptation, parental care behavior, scale‐eating, selfish shepherd effect

## Abstract

Alloparental care – care for unrelated young – is rare in animals, and its ecological or evolutionary advantages or, alternative maladaptive nature, remain unclear. We investigate alloparental care in the socially monogamous cichlid fish *Perissodus microlepis* from Lake Tanganyika that exhibits bi‐parental care. In a genetic parentage analysis, we discovered a surprisingly high percentage of alloparental care represented by brood mixing, extra‐pair paternity and extra‐pair maternity in all broods that we investigated. The percentage of nondescendant juveniles of other parents, i.e., brood mixing, ranged from 5% to 57% (mean = 28%). The distribution of genetic parentage also suggests that this socially monogamous species has, in fact, polygamous mating system. The prevalence of genetically mixed broods can be best explained by two, not mutually exclusive hypotheses on farming‐out and fostering behaviors. In the majority of broods, the sizes of the parents’ own (descendant) offspring were significantly larger than those of the adopted (nondescendant) juveniles, supporting the ‘selfish shepherd effect’ hypothesis, i.e., that foster parents preferentially accept unrelated “smaller or not larger” young since this would tend to lower the predation risks for their own larger offspring. There was also a tendency for larger parents particularly mothers, more so than smaller parents, to care predominantly for their own offspring. Larger parents might be better at defending against cuckoldry and having foreign young dumped into their broods through farming‐out behavior. This result might argue for maladaptive effects of allopatric care for the foster parents that only larger and possibly more experienced pairs can guard against. It needs to be determined why, apparently, the ability to recognize one's own young has not evolved in this species.

## Introduction

Parental care behavior evolves in response to the interplay of mating system, sexual selection, reproductive biology and ecology (e.g., Baylis 1981; Keenleyside 1991). Alloparental care – care for nondescendant young through brood mixing and/or extra‐pair matings (Wisenden 1999) – is a rare, but taxonomically widespread, phenomenon that is found in several groups of animals including mammals and birds (Riedman 1982), social insects (Hogendoorn et al. 2001) and also fishes (Wisenden 1999; Coleman and Jones 2011). Yet, the ecological circumstances that favor alloparental care and its evolutionary origins and benefits remain controversial since, obviously, investment in nondescendant young is expected to incur fitness costs on the caregiver while the potential fitness benefits often remain unclear (Clutton‐Brock 1991; Roldán and Soler 2011).

Fishes offer informative opportunities for the study of the evolution of parental care behaviors because of a wide range of their parental care patterns, and life history and reproductive features. However, it is often challenging to study parental care behaviors of fishes under natural conditions (Avise et al. 2002; Amundsen 2003; Coleman and Jones 2011). Molecular markers (e.g., microsatellites) made parentage analysis easier and provided intriguing insights into the parental care behaviors and mating systems in a number of fish species (Kellogg et al. 1995; DeWoody and Avise 2001; Avise et al. 2002; Sefc et al. 2008, 2012; Schaedelin et al. 2013). But, by comparison to other animals particularly birds, parentage studies of fishes are still relatively few, in particular in light of the large number of species and the enormous diversity of life history characteristics in this group (Avise et al. 2002; Amundsen 2003).

Six proximate mechanisms have been suggested to give rise to alloparental care in fishes (Wisenden 1999) – (1) brood farming out (Yanagisawa 1985) where parents ‘deliberately’ transfer their offspring to be cared for by other parents; (2) kidnapping (McKaye and McKaye 1977) where foster parents kidnap free‐swimming young of other parents; (3) independent offspring inclusion (Taborsky 1994) where deserted or stray juveniles join neighboring broods; (4) brood amalgamation (Eadie et al. 1988) where adjacent broods merge for cooperative care by more than one set of biological parents; (5) philopatric offspring (Taborsky and Limberger 1981) where offspring from previous breeding events stay at their natal territory and help their parents to nurse subsequent broods and (6) extension of alloparental care of eggs (Taborsky 1994). Still, the ultimate evolutionary origins and explanations as to the adaptive or nonadaptive, or maladaptive, natures as well as proximate mechanisms of alloparental care appear to vary at inter and, sometimes, even intraspecific levels (Sefc et al. 2009; Coleman and Jones 2011).

The evolution of alloparental care particularly in fishes might be best explained by the relatively low costs it entails to the foster parents, and the potentially high benefits it might confer (Wisenden 1999). Parental care in fishes usually does not involve feeding young, but primarily comprises only antipredatory behavior to protect the brood (Wisenden 1999) (but see Noakes and Barlow 1973; Noakes 1979). The presumed absence of additional energetic costs in fishes related to feeding young, as would be the case with most mammals and birds, may facilitate the evolution of alloparental care in fishes and it might evolve in response to strong selection through predation on young (McKaye 1981). Several ultimate explanations have been proposed to contribute to alloparental care evolution in fishes – including (1) the confusion effect (Taylor 1976) where predation success decreases with increasing brood size; (2) the dilution effect (McKaye and McKaye 1977) where per capita predation diminishes with increasing brood size; (3) the selfish herd effect (Mckaye et al. 1992) where a predation rate of young differs as a function of spatial variation in predation risk (Hamilton 1971); (4) the selfish shepherd effect (Wisenden and Keenleyside 1992) where antipredator competency varies among young in a mixed brood based on body size and (5) kin selection (Hamilton 1971). Evidence supporting the ‘selfish shepherd effect’ hypothesis, where body sizes of host (descendant) juveniles are predicted to be larger than (or at least similar to) those of adopted (nondescendant) juveniles, was demonstrated in Central American cichlids (genus *Cichlasoma*) (Noakes and Barlow 1973; Wisenden and Keenleyside 1992); however, such a pattern has, so far, not been found in Lake Tanganyikan cichlids (but see Ochi and Yanagisawa 1996). It seems reasonable to expect that larger parents would defend their broods more successfully against cuckoldry or dumping of unrelated young by other parents (relative to smaller parents), but in fishes, only few tests of this hypothesis have been carried out so far (e.g., Bisazza and Marconato 1988).

Prolonged bi‐parental or uniparental care of offspring, one of the peculiar life‐history characteristics of fishes in the family Cichlidae (Keenleyside 1991), might increase likelihood of the evolution of alloparental care. Nearly all of the more than 2000 species of East African cichlids (Turner et al. 2001) show brood care behavior – typically for 3–4 weeks or even longer (Keenleyside 1991), which would be expected to increase the chances of alloparental care. Several studies of cichlids have demonstrated the common occurrences of brood mixing caused by brood farming out [e.g., in the Lake Tanganyikan cichlids, *Perissodus microlepis* (Yanagisawa 1985), *Xenotilapia flavipinnis* (Yanagisawa 1986), and *Microdontochromis* sp. (Yanagisawa et al. 1996)], independent offspring inclusion (movements of free‐swimming fry) [in the Lake Malawi cichlids (Ribbink et al. 1980)], or either (or both) of these components [in the Lake Tanganyikan cichlid, *Neolamprologus caudopunctatus* (Schaedelin et al. 2013)]. The origins and mechanisms of alloparental care, however, appear to vary among cichlids as do parental brood care behaviors (Keenleyside 1991; Sefc 2011).

In addition, mating system types or mating behaviors are suggested to affect the degree and pattern of alloparental care in cichlids (Kuwamura 1986; Sefc 2011). For example, in maternally mouth‐brooding species where the mothers incubate eggs and fry in their buccal cavities, offspring often is fathered by multiple fathers (i.e., multiple paternity or extra‐pair paternity). In the very species‐rich haplochromine lineage of East African cichlids (>1800 species in this lineage alone) (Klett and Meyer 2002; Salzburger et al. 2005), multiple paternity appears to be relatively common and has been shown so far in, e.g., seven species of Lake Malawi cichlids including *Copadichromis cyclicos*,* Pseudotropheus* spp., *Melanochromis auratus* and *Protomelus* spp. (Kellogg et al. 1995) and the Lake Tanganyikan cichlid, *Ctenochromis horei* (Sefc et al. 2009). Surprisingly, however, the Tanganyikan maternal mouth‐brooder (*Simochromis pleurospilus*) showed the opposite pattern of elevated levels of multiple maternity (i.e., extra‐pair maternity) resulting from polygyny, with reduced multiple paternity (Sefc et al. 2012). Socially monogamous species typically form single pair bonds and usually also mate only with their partner as revealed by microsatellites, e.g., in mouth‐brooding cichlids, *Eretmodus cyanostictus* (Taylor et al. 2003) and *Xenotilapia rotundiventralis* (Takahashi et al. 2011), but since this is neither the case universally for substrate breeding cichlids, *Variabilichromis moorii* (Sefc et al. 2008) nor for *Amatitlania siquia* (Lee‐Jenkins et al. 2015), it appears that the relationship between genetic parentage and mating systems in cichlids does not always follow the expected pattern (Sefc 2011).

*Perissodus microlepis* is an ecologically highly specialized scale‐eating (lepidophagous) cichlid fish (Hori 1993; Lee et al. 2010, 2012, 2015; Kusche et al. 2012) endemic to Lake Tanganyika, Africa that inhabits the shallow rocky shore. This species is socially monogamous and its spawning typically takes place on a flat surface of projecting rocks. Females alone mouth‐brood eggs and embryos for 9–11 days and both parents then guard the free‐swimming juveniles together on the substrate for several weeks [i.e., bi‐parental brood‐carer (Yanagisawa 1985; H. J. Lee & A. Meyer, pers. obs.)]. During this guarding period, parents of *P. microlepis*, particularly males have been observed to farm out their fry to other conspecific (Yanagisawa 1985; Ochi et al. 1995; Ochi and Yanagisawa 2005) or even heterospecific breeding pairs (Ochi and Yanagisawa 1996). By conducting a cohort analysis of schooling juveniles of *P. microlepis* based on their size‐frequency distributions, it has been reported that 52 of 61 (85%) groups of schooling young presumably contained extra‐pair juveniles of other parents up to seven broods (Ochi et al. 1995). Yet, this reported level of brood mixing was inferred from body size measurements alone and genetic parentage analyses on broods of this species have not yet been undertaken.

We determined the degree and type of alloparental care in *Perissodus microlepis* by conducting genetic parentage analyses on broods and their guarding (fostering) breeding pairs. For a majority of broods, differences in body size between “descendant” and “nondescendant” young were assessed to test the predictions of the ‘selfish shepherd effect’ hypothesis. We predicted that body sizes of the former group are larger than (or at least similar to) those of the latter. The hypothesis that larger parents are better at defending their broods against cuckoldry or intrusion of unrelated young than smaller parents was also tested.

## Materials and Methods

### Sample collection

Juvenile *Perissodus microlepis* (*n *=* *481) were sampled from eight broods with their guarding breeding pairs (*n *=* *16) at Toby Veall's lodge (8°37.4′S, 31°12′E) in southern Lake Tanganyika, Zambia in April 2010 (Kusche et al. 2012; Lee et al. 2012). Sampling was conducted by diving with hand nets. We collected the broods and their guarding pairs when there were no neighboring *P. microlepis* fish adjacent to the sampling sites in order to avoid sampling artefacts. The number of samples collected per brood ranged from 8 to 113 (Table 1). Some of our sample sizes were smaller than the original brood size because it was not always possible to catch all the offspring in all broods, particularly if they were already free swimming. Body sizes (total length; TL) of the broods ranged from 7 to 15 mm, and those (standard length; SL) of the guarding male and female parents ranged from 8.3 to 10.4 cm and from 6.6 to 8.7 cm, respectively. Additionally, 30 adult fish were collected to assess marker polymorphism and allele frequencies in the study population. All samples were stored in 97% ethanol and vouchered in the fish collection at the University of Konstanz. The sex of the adult specimens was determined by examination of their gonads.

**Table 1:** Eight *Perissodus microlepis* broods with their foster (guarding) parents were sampled (Kusche et al. 2012). Level of alloparental care including brood mixing, extra‐pair paternity and extra‐pair maternity was estimated using two methods: (1) maximum likelihood implemented in COLONY 2.0 (Jones and Wang 2010) and (2) exclusion principle in FAP 3.6 (Taggart 2007) (the values in parentheses; ‘minimum’ level of alloparental care). The values in parentheses for number of full‐sib groups represent the full‐sib groups comprising more than one individual. Number of full‐sib groups in each type of alloparental care is shown in Table S2. In five broods analyzed, mean skull length of genetically assigned ‘descendant’ (host) juveniles was significantly larger than that of ‘nondescendant’ (adopted) juveniles (two‐way nested mixed‐model ANOVA; *F *=* *426.03, df = 9, *P *<* *0.001; see Fig. 3)

Brood number	Number of young in brood (*n*)	Proportion of alloparental care	Number of full‐sib groups	Mean skull length (mm) of ‘descendant’ juveniles (*n*)	Mean skull length (mm) of ‘nondescendant’ juveniles (*n*)
b1	28	0.61 (0.61)	13 (3)	2.62 ± 0.09 (11)	2.62 ± 0.14 (3)
b2	60	0.60 (0.60)	17 (3)	–	–
b3	90	1.00 (0.42)	16 (5)	–	–
b4	42	0.05 (0.05)	3 (1)	1.82 ± 0.04 (39)	1.79 ± 0.02 (2)
b5	113	0.22 (0.22)	15 (5)	3.44 ± 0.10 (70)	3.19 ± 0.29 (13)
b6	84	0.50 (0.46)	26 (5)	4.23 ± 0.13 (42)	3.81 ± 0.44 (31)
b7	56	0.32 (0.32)	13 (5)	2.47 ± 0.15 (33)	2.15 ± 0.44 (11)
b8	8	0.63 (0.63)	5 (2)	–	–
		Mean 0.49 (0.41)			

### Microsatellite genotyping

Genomic DNA was extracted from a small fraction of tissue from the posterior part of juveniles as well as from fin clips of the adults, and genotyped with six polymorphic microsatellite loci – UNH2101 (Stewart and Albertson 2010), Abur25, Abur44, Abur61, Abur98 and Abur117 (Sanetra et al. 2009) – which had been used in our previous population genetics study of *P. microlepis* (Lee et al. 2010). Forward primers were labeled with a fluorescent dye (6‐FAM or HEX). PCR reaction was carried out in 10 *μ*L volumes (1× PCR buffer, 25 *μ*mol/L of each dNTP, 0.5 *μ*mol/L of each of the forward and reverse primers, 0.1 U Taq polymerase [Life Technologies, Darmstadt, Germany] and 30–50 ng of DNA). Some juvenile samples were failed to be successfully amplified at one or two loci (Table S1). PCR products were diluted in formamide HiDi and electrophoresed in an ABI 3130*xl* automated sequencer (Applied Biosystems, Darmstadt, Germany). Fragment sizes were compared to ROX 500 bp size standard (Applied Biosystems) as determined using GeneMapper software 4.0 (Applied Biosystems).

### Parentage analyses

Using the population sample combined with 16 foster parents (*n *=* *46), marker polymorphism and HWE (Hardy─Weinberg Equilibrium) were assessed in GENEPOP 4.0 (Rousset 2008). Frequency of null alleles was estimated in IDENTITY 4.0 (Sefc and Wagner 1999) based on the difference between expected (*H*_*E*_) and observed (*H*_*O*_) heterozygosities as suggested in Brookfield 1996). Two exclusion probabilities – (1) one parent known and (2) neither parent known – were calculated for each locus as well as for all loci combined in GERUD 2.0 (Jones 2005) according to Dodds et al. (1996). Exclusion probability represents the likelihood that an unrelated candidate parent (that is randomly chosen from a population) would be eliminated from consideration as a true parent by the locus of interest (Chakraborty et al. 1988).

Parentage analyses were performed with two approaches – (1) exclusion principle using FAP 3.6 (Taggart 2007) and (2) maximum likelihood using COLONY 2.0 (Jones and Wang 2010). According to the exclusion method, at least one allele should be shared by a parent and an offspring at a codominant microsatellite locus following Mendelian inheritance. FAP uses this principle to make assignments of offspring to parent‐pairs using pair‐wise approaches. By comparison, COLONY applies full‐pedigree based maximum likelihood to simultaneously assign both sib‐ship and parentage relationships among all sampled individuals jointly, which allows higher statistical power and thus more accurate results than pair‐wise and exclusion approaches (Wang and Santure 2009; Wang 2012). Analyses with COLONY were first run with setting a genotyping error rate of 10% to identify potential errors as suggested in previous studies (Sefc et al. 2008, 2012). No scoring errors were found, such that any possible scoring errors should be due to mutations, whose frequency is, in principle, presumed to be low. Error rate was therefore set to zero for final analyses (Table S1). Moreover, by doing replicate PCRs, we further confirmed genotypes of the 16 foster parents and juvenile individuals that turned out to be young of alloparental care at each of the six loci.

To test the hypothesis that the larger and presumably older and more experienced parents of *P. microlepis* are more successful in brood defense against cuckoldry or dumping of foreign young, a correlation analysis was conducted to test for a significant relationship between body size (standard length; SL) of male and female parents and proportion of their own juveniles. Juveniles of within‐pair plus extra‐pair maternity or paternity were considered his or her own offspring, respectively.

### Analysis of differences in body size between descendant and nondescendant juveniles

To test the ‘selfish shepherd effect’ hypothesis (Wisenden and Keenleyside 1992), differences in body size were analyzed between two groups of genetically assigned descendant and nondescendant juveniles for five out of our eight broods (b1, 4, 5, 6, 7) that had sufficient sample sizes. For this analysis, the head of each juvenile was cleared and double‐stained following Walker and Kimmel (2007) (Lee et al. 2015). Skull length of the stained specimens was then measured as a proxy for the size in ImageJ 1.45r (http://imagej.nih.gov/ij) from standardized photographs in a lateral view with an implemented scale (Fig. S1), as skull length correlates tightly with body size (Copp and Kováč 2003; H. J. Lee, V. Heim & A. Meyer, pers. obs.). The data were then analyzed in a two‐way ‘nested’ mixed‐model ANOVA; the ‘genetic status’ of juveniles (e.g., “descendant” or “nondescendant”) was considered as a ‘fixed’ effect and ‘brood’ as a random effect. Juveniles that were descendants of “both” parents were considered “descendant”, and juveniles that were not descendants of “either” parent (i.e., brood mixing) were considered “nondescendants”, as suggested in Wisenden and Keenleyside (1992).

## Results

### Parentage analyses

The six microsatellite loci genotyped were polymorphic; expected (*H*_E_) and observed (*H*_O_) heterozygosities averaged across the loci in the study population (Toby Veall's lodge) were 0.787 and 0.834, respectively with the number of alleles per locus ranging from 8 to 15 (mean number = 11; Table 2). The population conformed to HWE expectations at all loci, which had previously been indicating no significant association of alleles among those loci (i.e., no linkage disequilibrium) (Lee et al. 2010). The estimated frequencies of null alleles at the loci were close to zero (Table 2), indicating there is very low probability for null alleles. Combined across the loci, the exclusion probability of both cases – one parent known and neither parent known – was 99.6% and 96.7%, respectively (Table 2).

**Table 2:** Summary of genetic diversity statistics for six microsatellite loci of the 46 population samples that were genotyped in the present study. *H*_E_: expected heterozygosity; *H*_O_: observed heterozygosity; *P*_HWE_: probability of departure from Hardy–Weinberg Equilibrium; *F*(0): estimated frequency of null alleles; *E*_1_: exclusion probability when one parent is known; *E*_2_: exclusion probability when neither parent is known

Locus	Number of alleles	Range of allele frequencies	*H*_E_	*H*_O_	*P*_HWE_	*F*(0)	*E*_1_	*E*_2_
Abur25	8	0.011–0.359	0.733	0.783	0.837	−0.033	0.485	0.313
Abur44	12	0.011–0.500	0.716	0.826	0.561	−0.069	0.521	0.332
Abur61	12	0.011–0.250	0.852	0.761	0.127	0.044	0.693	0.527
Abur98	11	0.011–0.402	0.748	0.870	0.352	−0.074	0.536	0.358
Abur117	15	0.011–0.228	0.885	0.913	0.998	−0.020	0.753	0.603
UNH2101	8	0.011–0.326	0.788	0.848	0.635	−0.038	0.573	0.394
All loci	Mean 11		Mean 0.787	Mean 0.834	0.733		0.996	0.967

**Figure 1:**
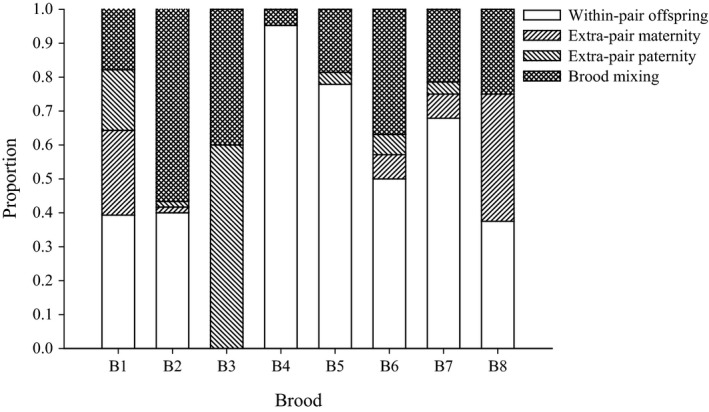
Proportion of alloparental care in *Perissodus microlepis* with bi‐parental care and social monogamy, as revealed by maximum likelihood method in COLONY (Jones and Wang 2010). White bar (within‐pair offspring): genetically assigned ‘descendant’ juveniles of both parents; right‐upward diagonal‐line bar (extra‐pair maternity): juveniles of multiple maternity resulting from polygyny; left‐upward diagonal‐line bar (extra‐pair paternity): juveniles of multiple paternity resulting from polyandry; crosses bar (brood mixing): ‘nondescendant’ juveniles to either parent.

The analyses of eight *P. microlepis* broods revealed unexpectedly high levels of alloparental care via brood mixing and extra‐pair matings (such as extra‐pair [multiple] paternity and extra‐pair [multiple] maternity), ranging from 5% (b4) to 63% (b8) based on exclusion principle and from 5% (b4) to 100% (b3) based on maximum likelihood, respectively (Table 1; Fig. 1). Note that these values represent proportion of juveniles within each of the broods. The discrepancy between the two methods concerning the level of alloparental care was due to underestimates or overestimates of the number of adopted juveniles for b3 from exclusion or maximum likelihood approaches (Table 1). Except that brood, results of genetic parentage were almost identical to each other for the remaining broods (see Table 1).

Not only brood mixing (unrelated juveniles of other parents), but also extra‐pair paternity and also extra‐pair maternity were all found in our sample, based on maximum likelihood estimations (Fig. 1). Percentage of brood mixing that would be expected to result from brood farming out was the greatest, ranging from 4.8% (b4) to 56.7% (b2) (mean = 27.7%) (Fig. 1). The proportion of extra‐pair paternity ranged from zero (b4, 8) to 60% (b3) (mean across the broods = 11.6%), and extra‐pair maternity ranged from zero (b3, 4, 5) to 37.5% (b8) (mean = 9.8%). Unexpectedly, the eight broods turned out to be composed of three (b4) up to 26 (b6) full‐sib groups, although a majority of the observed full‐sib groups were represented in only a single individual (Table 1). The major full‐sib group (the largest in number) within broods always corresponded to descendant offspring of foster parents except b3. Detailed information on the distribution of parentage (e.g., number of full‐sib groups [number of parent‐pairs]) in each type of alloparental care (brood mixing, extra‐pair paternity and extra‐pair maternity) across the eight broods is shown in Table S2.

**Figure 2:**
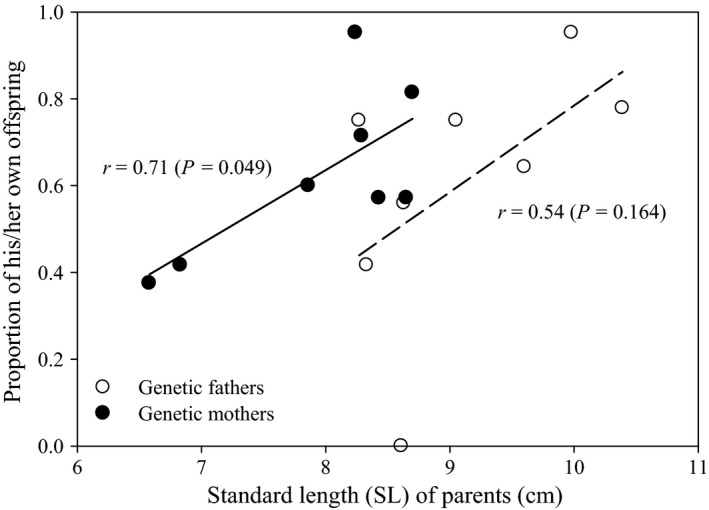
Larger parents of *Perissodus microlepis* tend to have higher proportions of their own young than smaller parents, although this trend is statistically significant only for mothers (*r *=* *0.71, *n *=* *8, *P *=* *0.049). Unfilled circles: genetic fathers; filled circles: genetic mothers.

A significant positive correlation was detected between the female parents’ body size (SL) and proportion of her ‘descendant’, i.e., own, offspring (*r *=* *0.71, *n *=* *8, *P *=* *0.049; Fig. 2), suggesting that the larger mothers of *P. microlepis* more often cared predominantly for their own offspring. This relationship was, however, not statistically significant for fathers (*r *=* *0.54, *n *=* *8, *P *=* *0.164), in spite of the similar trend detected (Fig. 2).

### Size differences between descendant and nondescendant juveniles

**Figure 3:**
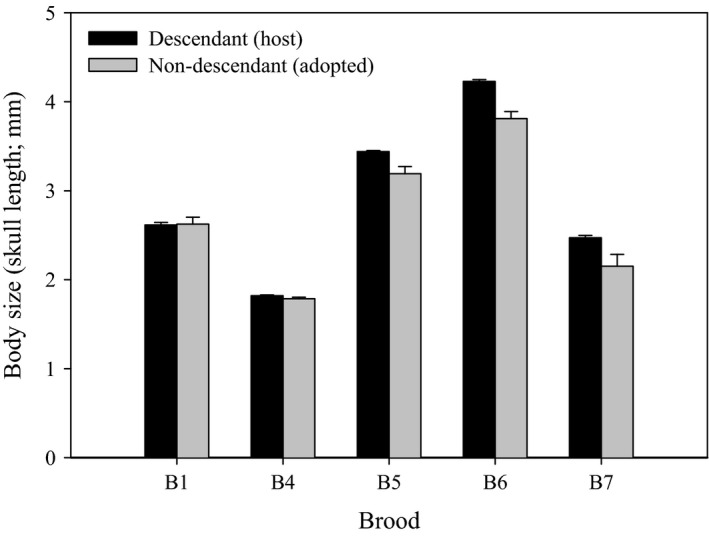
Significant differences in body size between two groups of genetically descendant (host; black bars) and nondescendant (adopted; gray bars) juveniles in five broods of *Perissodus microlepis* (two‐way nested mixed‐model ANOVA;* F *=* *426.03, df = 9, *P *<* *0.001; see Table 1). Skull length was measured as a proxy for the size of every juvenile fish (*n *=* *255) (Fig. S1). The data support the ‘selfish shepherd effect’ hypothesis that the sizes of descendant young are significantly larger than (or at least similar to) those of nondescendant young (Wisenden and Keenleyside 1992). The error bars indicate SE (standard error) of the mean.

In five broods examined, genetically descendant (host) juveniles of foster parents were significantly larger compared to the nondescendant (adopted) juveniles of other parents (two‐way nested mixed‐model ANOVA; *F *=* *426.03, df = 9, *P *<* *0.001; Table 1; Fig. 3), supporting the ‘selfish shepherd effect’ hypothesis. Four of the five broods (b4, 5, 6, 7) exhibited larger mean skull length (mm) of ‘descendant’ juveniles than ‘nondescendant’ ones, whereas b1 showed no size difference between the two groups (descendant [*n *=* *11]: 2.62 ± 0.09 [SD] *mm*, nondescendant [*n *=* *13]: 2.62 ± 0.14; Table 1; Fig. 3).

## Discussion

The evolution of alloparental care raises evolutionarily interesting questions since at first glance it would appear to be a ‘maladaptive’ behavior (Wisenden 1999). However, evidence is accumulating that alloparental care can actually increase the fitness of alloparents through (1) protection of their young against predation (Wisenden 1999) or (2) mating benefits [e.g., female mating preference for alloparental males (Stiver and Alonzo 2011)]. The prevalence of alloparental care in the Lake Tanganyikan scale‐eating cichlid fish, *Perissodus microlepis* found here exceeds all reports so far documented for any other cichlids. Brood mixing and extra‐pair matings here are not only exceptionally common, but also multifaceted (e.g., farming out, polygamy; see below). Our data support the ‘selfish shepherd effect’ hypothesis that the alloparents of *P. microlepis* might receive fitness benefits by diluting the predation risks on their own offspring by preferentially accepting unrelated fry at smaller body sizes than (or at least similar to) their own young (Noakes and Barlow 1973; Bisazza and Marconato 1988; Wisenden and Keenleyside 1992). Although the number of parental fish analyzed in this study is small (*n *=* *8; each for female or male parents), our results show that larger parents, especially mothers, have higher proportions of their own offspring. This trend may suggest that larger mothers are better at defending against cuckoldry and having foreign young dumped into their broods. It would also be conceivable that larger mothers who perhaps produce more offspring (i.e., higher fecundity) than smaller ones would have proportionally less nondescendant juveniles. This would also explain the lack of a correlation for male parents’ body size since the number of offspring is solely dependent on the number of eggs laid by mothers.

### High levels of alloparental care

We find that alloparental care is particularly common in this species – we found extra‐pair fry in all eight broods analyzed. On average, approximately 28% of all young in broods (ranging from 4.8% to 56.7%) resulted potentially from brood farming‐out behavior of *P. microlepis* (Table 1; Fig. 1). This is a higher level than previously reported based on size measurements alone (Ochi et al. 1995). This difference is most likely due to a systematic underestimating of numbers of adopted juveniles by measuring size differences among small fry in the previous study (Ochi et al. 1995). Alternatively, population density was considerably higher in our sampling locality (Toby Veall's lodge) compared to those of the previous study (Ochi et al. 1995) and this would be expected to result in more active nests in the neighborhood, which possibly leads to higher levels of brood mixing through movements of free‐swimming fry in our study population (Sefc et al. 2008).

The elevated levels of brood mixing in *P. microlepis* can be again best explained by the reported farming‐out behavior (Yanagisawa 1985) since unrelated juveniles of both parents within broods (brood mixing shown in Fig. 1), which would be anticipated to result from farming out, make up the largest proportion of alloparental care. An alternative hypothesis would be that some of the juveniles independently move and join broods guarded by fostering parents [i.e., independent offspring inclusion (Taborsky 1994)], which has previously been documented in the Lake Malawi cichlid species, *Haplochromis chrysonotus* (Ribbink et al. 1980). Kidnapping would also possibly account for the observed patterns of the prevalence of brood mixing, as previously reported in convict cichlids (e.g., McKaye and McKaye 1977; Wisenden and Keenleyside 1992). Nevertheless, farming‐out behavior seems to be the most plausible mechanism for alloparental care in *P. microlepis*, although the fairly moderate proportions of offspring of extra‐pair paternity (mean = 11.6%) as well as of extra‐pair maternity (mean = 9.8%) were also detected in this species.

Previous field observations of farming‐out behavior in *P. microlepis* agree with our genetic data on the common occurrences of unrelated juveniles of other parents within broods (Yanagisawa 1985; Ochi and Yanagisawa 2005). Scuba observations for 2 months at breeding sites found that 90 of 108 broods (83%) ultimately vanished during that time, although their body size was certainly not sufficiently large enough to become independent (25–31 mm in SL) (Yanagisawa 1985). This is most likely due to parents’ farming out their broods to one another until only a few remained (Ochi and Yanagisawa 2005). Alternatively, it is also possible that predation accounts for the disappearance of premature young. The farming‐out behavior was actually observed in 32% of breeding pairs when, mostly male, parents took young into their mouth and travelled along the shore, sometimes more than a hundred meters, and deserted them under schools (one to three) of other parents (Ochi and Yanagisawa 2005). Moreover, the presence of obviously different size (age) classes within broods further supports farming‐out behavior in this species (Ochi et al. 1995; Ochi and Yanagisawa 1996; H. J. Lee, V. Heim & A. Meyer, pers. obs.). Observations of mixed broods, most likely due to brood farming out, have also been documented in several other Lake Tanganyikan cichlids with bi‐parental care both within and even across species (including in *Lepidiolamprologus elongates*,* L. attenuates*,* Neolamprologus caudopunctatus*,* N. tetracanthus* and *Haplotaxodon microlepis*) [Ochi and Yanagisawa 1996); reviewed in Sefc 2011)].

The ecological circumstances and the evolutionary fitness advantages of brood farming out in *P. microlepis* remain to be documented. The observed male‐biased farming‐out behavior would be expected to shorten the times between remating (Ochi and Yanagisawa 2005). We find that larger fathers of *P. microlepis* appear to possess higher portions of their own young (Fig. 2), although this trend was not statistically significant, and this is possibly caused by being more successful at defending against having foreign young added to broods (farming out) guarded by larger (older) compared to smaller (younger) fathers. However, this hypothesis remains to be tested in further studies on *P. microlepis*. It can be done with laboratory‐based “juvenile (fry) transfer experiments” to determine if larger fathers tend to better defend their young, compared to smaller ones, when encountering foreign young (e.g., Wisenden and Keenleyside 1992).

The observed extra‐pair paternity and maternity also indicates that the mating system in this socially monogamous species is actually polygamy. Polyandry in *P. microlepis* is not very surprising given that this mating system is widespread among maternally mouth‐brooding species of cichlids [e.g., the Tanganyikan cichlids *Ctenochromis horei* (Sefc et al. 2009)]. Extra‐pair paternity (resulting from polyandry) can be attributed to male sneaking activities, or from female soliciting followed by spawning with visiting extra‐pair males (Kuwamura 1986). Polygyny is relatively rare in mouth‐brooding cichlids (not substrate breeders), particularly socially monogamous species, but was recently found in the Tanganyikan maternally mouth‐brooding species, *Simochromis pleurospilus* (Sefc et al. 2012). Sefc et al. (2012) suggested that allomaternal care in *S. pleurospilus* may evolve as it can provide selective advantages for the “wrong” mother indirectly through reduction in brood predation if she is genetically related to the “right” parents. Multiple maternity may also occur if active free‐swimming young intrudes from neighboring territories where the fish are close relatives (Sefc et al. 2012) or where broods have been sired by the resident male when female carries the eggs in her mouth. ‘Male‐territory‐visiting‐polygyny’ (Kuwamura 1986) can explain the observed extra‐pair maternity in *P. microlepis* with maternally mouth‐brooding of eggs and small fry followed by prolonged biparentally guarding of free‐swimming fry. It would be conceivable that extra‐pair females visit the nest and spawn with the nest resident male. We also find that the degree of extra‐pair paternity and extra‐pair maternity varies across the eight families examined (Fig. 1). However, it is uncertain what ecological factors drive this among‐pairs variability in the amount of polygamy, but this issue would be warranted for future research avenue.

The observed high number of full‐sib groups within broods (e.g., distribution of young across many different parents; Tables 1, S2) was possibly due to overestimation of maximum likelihood. Using simulated data, Sefc and Koblmüller (2009) suggested that maximum likelihood in COLONY performs reliably with respect to parent number estimates (e.g., number of full‐sib groups), if ≥5~7 microsatellite markers of *H*_E_ ≥ 0.84 were to be used, highlighting the significance of marker polymorphism. The six markers used in our study showed mean *H*_E_ = 0.787, slightly lower than the values recommended, which may result in overestimation from COLONY (Sefc and Koblmüller 2009). Nevertheless, our exclusion probability estimates (one parent known: 99.6%, neither parent known: 96.7%) seem to argue against this possibility. However, the major full‐sib group (the largest in number) within broods always belongs to descendant offspring of foster parents (except b3 that did not hold any host juveniles).

### Selfish shepherd effect – is accepting smaller adopted young beneficial?

Our data best support the ‘selfish shepherd effect’ hypothesis (Wisenden and Keenleyside 1992), according to which non‐descendant young are selectively accepted by host parents if they are not larger in body size than the host's young (Noakes and Barlow 1973). This strategy evolves because host parents might increase their fitness by reducing the predation risks on their own offspring, since larger juveniles might have better anti‐predator competency than smaller juveniles (Wisenden and Keenleyside 1994). Although only five broods of *P. microlepis* could be examined in this regard, this pattern was apparent. Using juvenile transfer experiments with smaller or larger foreign young than host young, these predictions could also be tested further in the laboratory to determine whether the size difference between host and foreign juveniles will be maintained by discrimination on the part of host parents, as suggested in Wisenden and Keenleyside 1992).

We find that the socially monogamous cichlid *Perissodus microlepis* might actually be an effectively polygamous species, and that it often farms out its brood to other caring pairs. The occurrence of alloparental care has been reported for this species repeatedly, but our study is the first to demonstrate the prevalence of alloparental care – brood mixing, extra‐pair paternity and maternity – using genetic parentage analysis. The best hypotheses that would explain this unusual behavior are the ‘selfish shepherd effect’ and possibly time to remating constraints in particular on males that farm out part of their broods particularly frequently.

## Data Accessibility

The microsatellite data for the population samples and morphological data (e.g., body sizes of parents and skull length of descendant and nondescendant juveniles) have been deposited in DRYAD, entry doi:10.5061/dryad.rj408.

## Conflict of Interest

None declared.

## Supporting information


**Figure S1.** Body size of every juvenile fish (*n *=* *255) from five broods (b1, 4, 5, 6, 7) of *Perissodus microlepis* was approximated by estimating ‘skull length’ indicated in a dotted horizontal line.

**Table S1.** Raw dataset (the COLONY input files) of six microsatellites including data on allele frequencies, marker type error rates, female parent genotypes, male parent genotypes and offspring genotypes. Parental fish ID is as follows: b1 (10995: male parent [M], 10996: female parent [F]), b2 (11001 [M], 11002 [F]), b3 (11015 [M], 11016 [F]), b4 (11025 [M], 11026 [F]), b5 (11031 [M], 11032[F]), b6 (11040 [M], 11039 [F]), b7 (11043 [M], 11044 [F]), and b8 (11035 [M], 11036 [F]).

**Table S2.** Detailed information on the distribution of parentage (e.g., number of full‐sib groups [number of parent‐pairs]) in each type of alloparental care (brood mixing, extra‐pair paternity [multiple paternity], and extra‐pair maternity [multiple maternity]) across the eight *Perissodus microlepis* broods using maximum likelihood method implemented in COLONY 2.0 (Jones and Wang 2010).
