# Supplementary material for: Genetic evidence for prevalence of alloparental care in a socially monogamous biparental cichlid fish, Perissodus microlepis, from Lake Tanganyika supports the “selfish shepherd effect” hypothesis
Source: Ecol Evol. 2016 Mar 21;6(9):2843–53. doi: 10.1002/ece3.2089 (PMC4863010; doi:10.1002/ece3.2089)
Supplement: Supplementary file 1 — Figure S1. Body size of every juvenile fish (n = 255) from five broods (b1, 4, 5, 6, 7) of Perissodus microlepis was approximated by estimating ‘skull length’ indicated in a dotted horizontal line. [file ECE3-6-2843-s001.docx]

**Supporting Information Fig. S1.** Body size of every juvenile fish (*n* = 255) from five broods (b1, 4, 5, 6, 7) of *Perissodus microlepis* was approximated by estimating ‘skull length’ indicated in a dotted horizontal line. Two landmarks including – the most anterior point of the premaxillary bone of upper jaw (filled black circle ‘a’) and the most anterior point of vertebrae (‘b’) – were digitized to estimate the skull length. The bar denotes a scale of 1 mm.


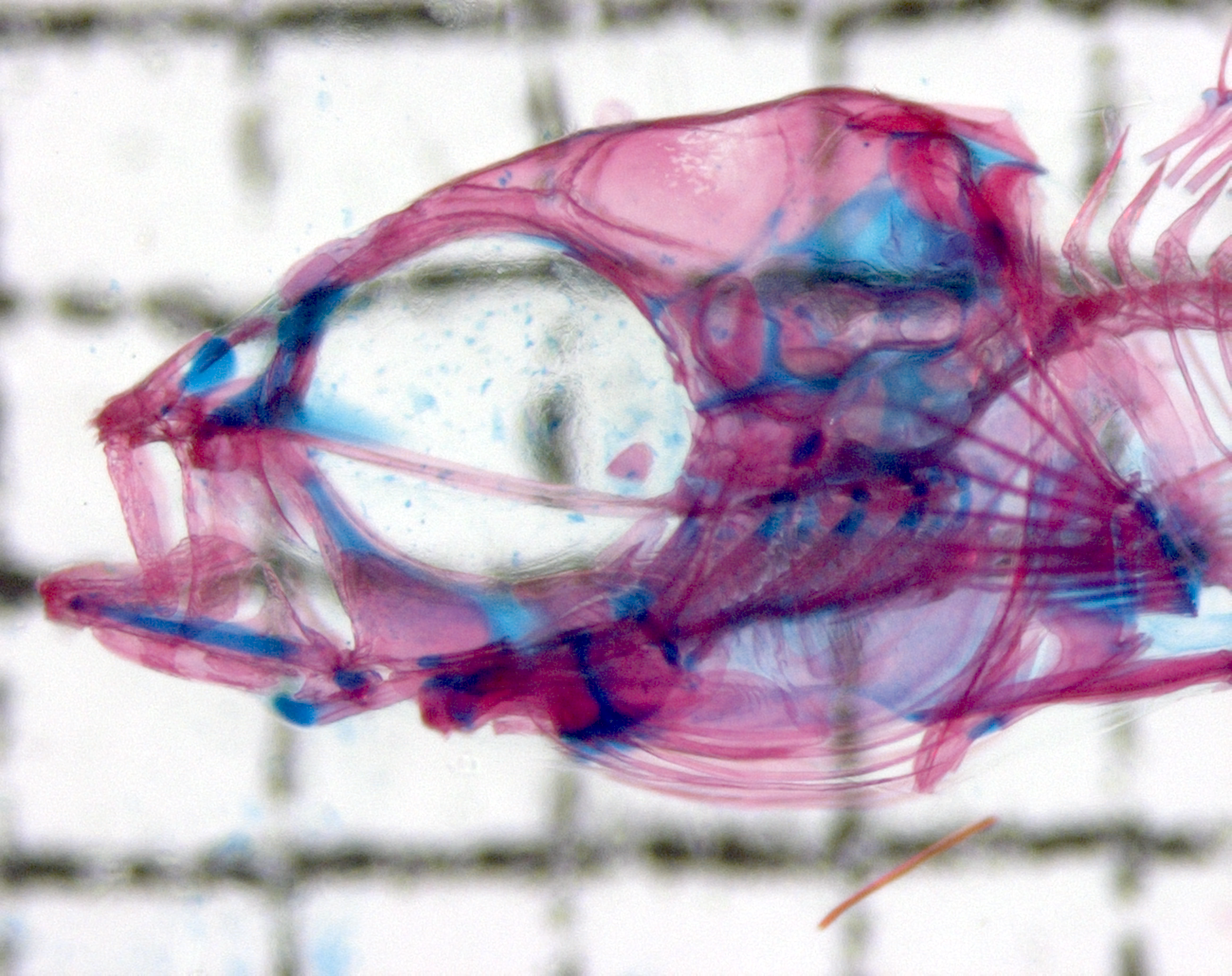


**b**

**a**
