# Supplementary material for: Genetic evidence for prevalence of alloparental care in a socially monogamous biparental cichlid fish, Perissodus microlepis, from Lake Tanganyika supports the “selfish shepherd effect” hypothesis
Source: Ecol Evol. 2016 Mar 21;6(9):2843–53. doi: 10.1002/ece3.2089 (PMC4863010; doi:10.1002/ece3.2089)
Supplement: Supplementary file 3 — Table S2. Detailed information on the distribution of parentage (e.g., number of full‐sib groups [number of parent‐pairs]) in each type of alloparental care (brood mixing, extra‐pair paternity [multiple paternity], and extra‐pair maternity [multiple maternity]) across the eight Perissodus microlepis broods using maximum likelihood method implemented in COLONY 2.0 (Jones and Wang 2010). [file ECE3-6-2843-s003.docx]

**Supporting Information Table S2.** Detailed information on the distribution of parentage (e.g. number of full-sib groups [number of parent-pairs]) in each type of alloparental care (brood mixing, extra-pair paternity [multiple paternity], and extra-pair maternity [multiple maternity]) across the eight *Perissodus microlepis* broods using maximum likelihood method implemented in COLONY 2.0 (Jones and Wang 2010).

| Brood number | Number of young in brood (*n*) | Proportion of alloparental care^a^ | Total number of full-sib groups | Number of full-sib groups for brood mixing | Number of full-sib groups for extra-pair paternity | Number of full-sib groups for extra-pair maternity |
| --- | --- | --- | --- | --- | --- | --- |
| b1 | 28 | 0.61 (0.61)^b^ | 13 (3)^c^ | 5 (5)^d^ | 2 (5)^d^ | 5 (7)^d^ |
| b2 | 60 | 0.60 (0.60) | 17 (3) | 14 (34) | 1 (1) | 1 (1) |
| b3 | 90 | 1.00 (0.42) | 16 (5) | 13 (36) | 3 (54) | - |
| b4 | 42 | 0.05 (0.05) | 3 (1) | 2 (2) | - | - |
| b5 | 113 | 0.22 (0.22) | 15 (5) | 10 (21) | 4 (4) | - |
| b6 | 84 | 0.50 (0.46) | 26 (5) | 15 (31) | 5 (5) | 5 (6) |
| b7 | 56 | 0.32 (0.32) | 13 (5) | 8 (12) | 1 (2) | 3 (4) |
| b8 | 8 | 0.63 (0.63) | 5 (2) | 2 (2) | - | 2 (3) |
|  |  | Mean 0.49 (0.41) |  |  |  |  |

^a^ Alloparental care comprises brood mixing, extra-pair paternity and extra-pair maternity.

^b^ Values in parentheses denote ‘minimum’ level of alloparental care estimated from exclusion principle in FAP 3.6 (Taggart 2007).

^c^ Values in parentheses denote number of the full-sib groups comprising more than one individual.

^d^ Values in parentheses denote number of individuals that belong to the respective categories of alloparental care (brood mixing, extra-pair paternity, extra-pair maternity) in each brood.
